# Supplementary material for: Population genomics provides insights into the evolution and adaptation to humans of the waterborne pathogen Mycobacterium kansasii
Source: Nat Commun. 2021 May 3;12:2491. doi: 10.1038/s41467-021-22760-6 (PMC8093194; doi:10.1038/s41467-021-22760-6)
Supplement: Supplementary file 1 — Supplementary Information [file 41467_2021_22760_MOESM1_ESM.pdf]

Population genomics provides insights into the evolution and adaptation to humans of the waterborne pathogen *Mycobacterium kansasii*

Tao Luo*, Peng Xu, Yangyi Zhang, Jessica L. Porter, Marwan Ghanem, Qingyun Liu, Yuan Jiang, Jing Li, Qing Miao, Bijie Hu, Benjamin P. Howden, Janet A. M. Fyfe, Maria Globan, Wencong He, Ping He, Yiting Wang, Houming Liu, Howard E. Takiff, Yanlin Zhao*, Xinchun Chen*, Qichao Pan*, Marcel A. Behr*, Timothy P. Stinear*, Qian Gao*

- **Supplementary Figures 1-16**
- **Supplementary Tables 1, 2**
- **Supplementary References**

*** Correspondence to:**

Qian Gao (qiangao@fudan.edu.cn)

Timothy P. Stinear (tstinear@unimelb.edu.au)

Marcel A. Behr (marcel.behr@mcgill.ca)

Qichao Pan (panqichao@scdc.sh.cn)

Xinchun Chen (chenxinchun@szu.edu.cn)

Yanlin Zhao (zhaoyl@chinacdc.cn)

Tao Luo (taoluo@scu.edu.cn)

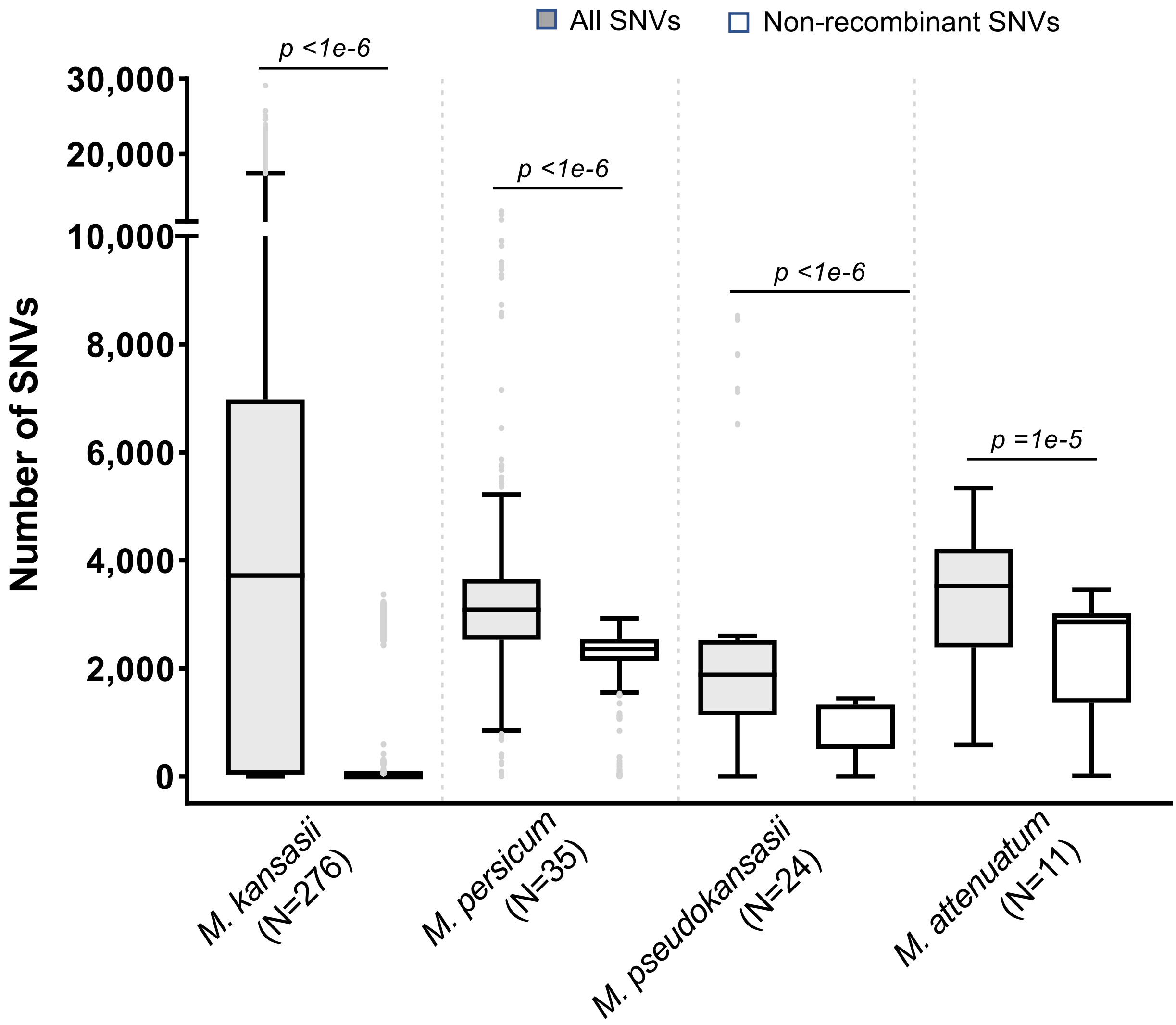

Supplementary Fig. 1. Pairwise difference of core genome single nucleotide variation (SNV) in the four major MKC species. All SNVs, SNVs from spontaneous mutations or recombinations; Non-recombinant SNVs, SNVs from spontaneous mutations. Boxes show the median and interquartile range (IQR) while whiskers extend to a maximum of 1.5× IQR. Significance level between the distances of all and non-recombinant SNVs for each species is determined by the Mann Whitney test (two-sided). Source data are provided as a Source Data file.

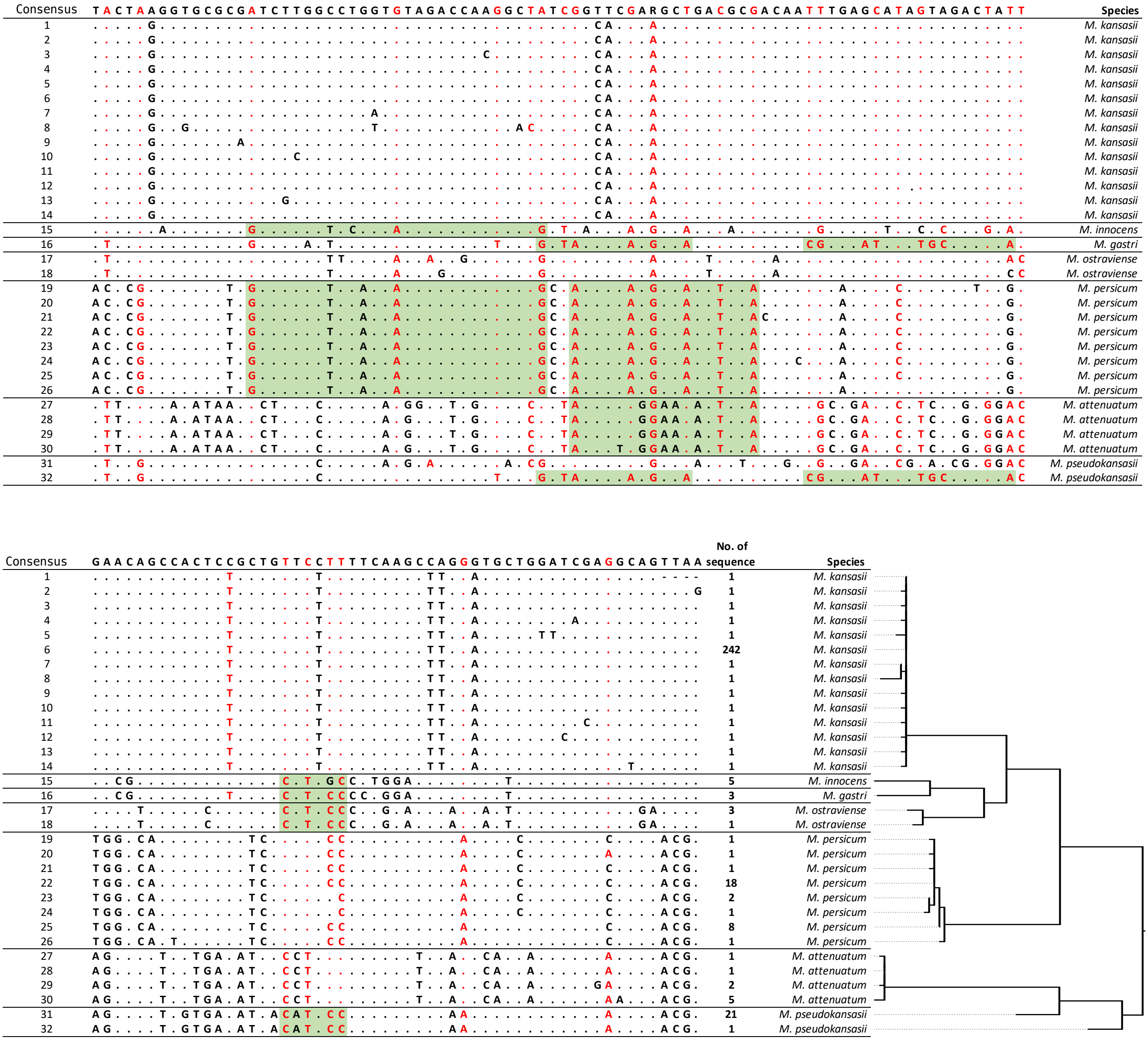

Supplementary Fig. 2. The neighbor-joining phylogeny and the variable loci of the 16S-ITS-23S rRNA alignments. Dot, same base as the consensus; Red base, homoplastic loci; green band, region of potential recombination.

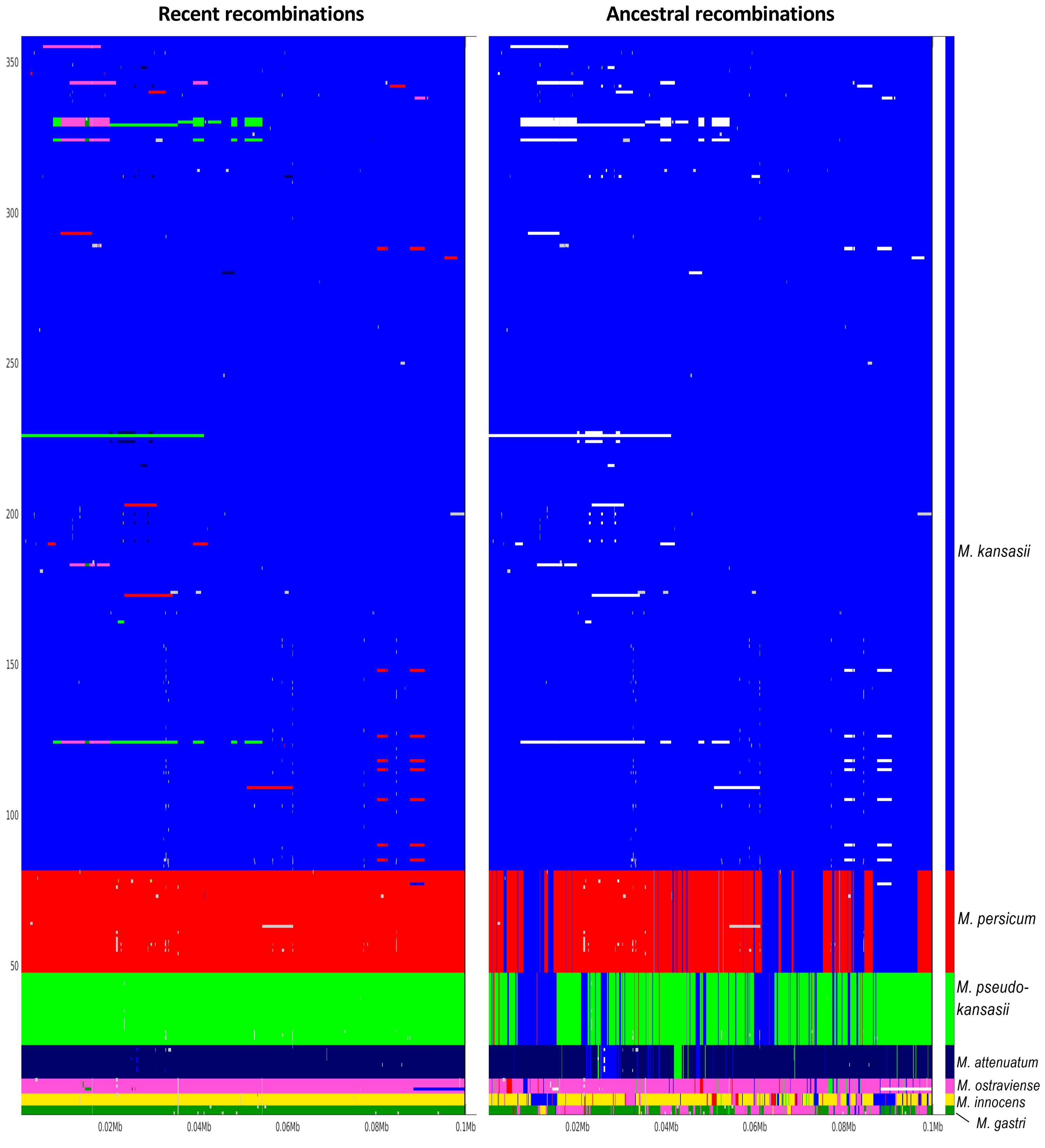

Supplementary Fig. 3. Genomic mosaics due to recent (left) and ancestral (right) recombinations between MKC species in a 100,000 bp region of the core genome. Each panel shows a multiple alignment of 358 sequences with colors representing ancestral sequence patterns of the MKC species as defined on the far left. The white lines in the right panel correspond to the recent recombinant fragments in the left.

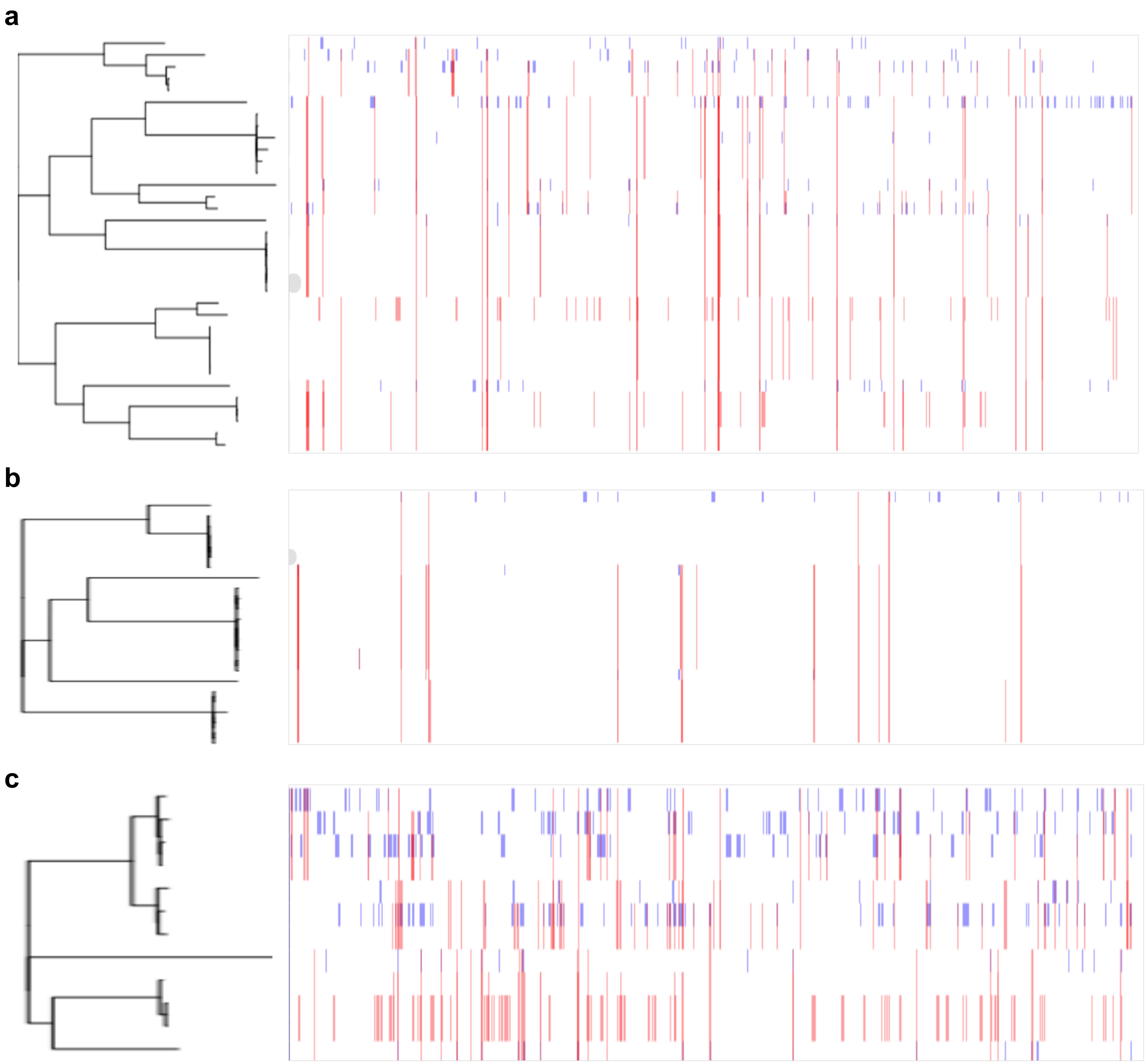

Supplementary Fig. 4. Genomic recombinations in *M. persicum* (**a**), *M. pseudokansasii* (**b**) and *M. attenuatum* (**c**) predicted by Gubbins. Left panel, maximum-likelihood phylogenies based on SNVs in non-recombinant genomic regions. Right panel, genomic recombinations. Blue blocks, unique recombinant segments; Red blocks, shared recombinant segments.

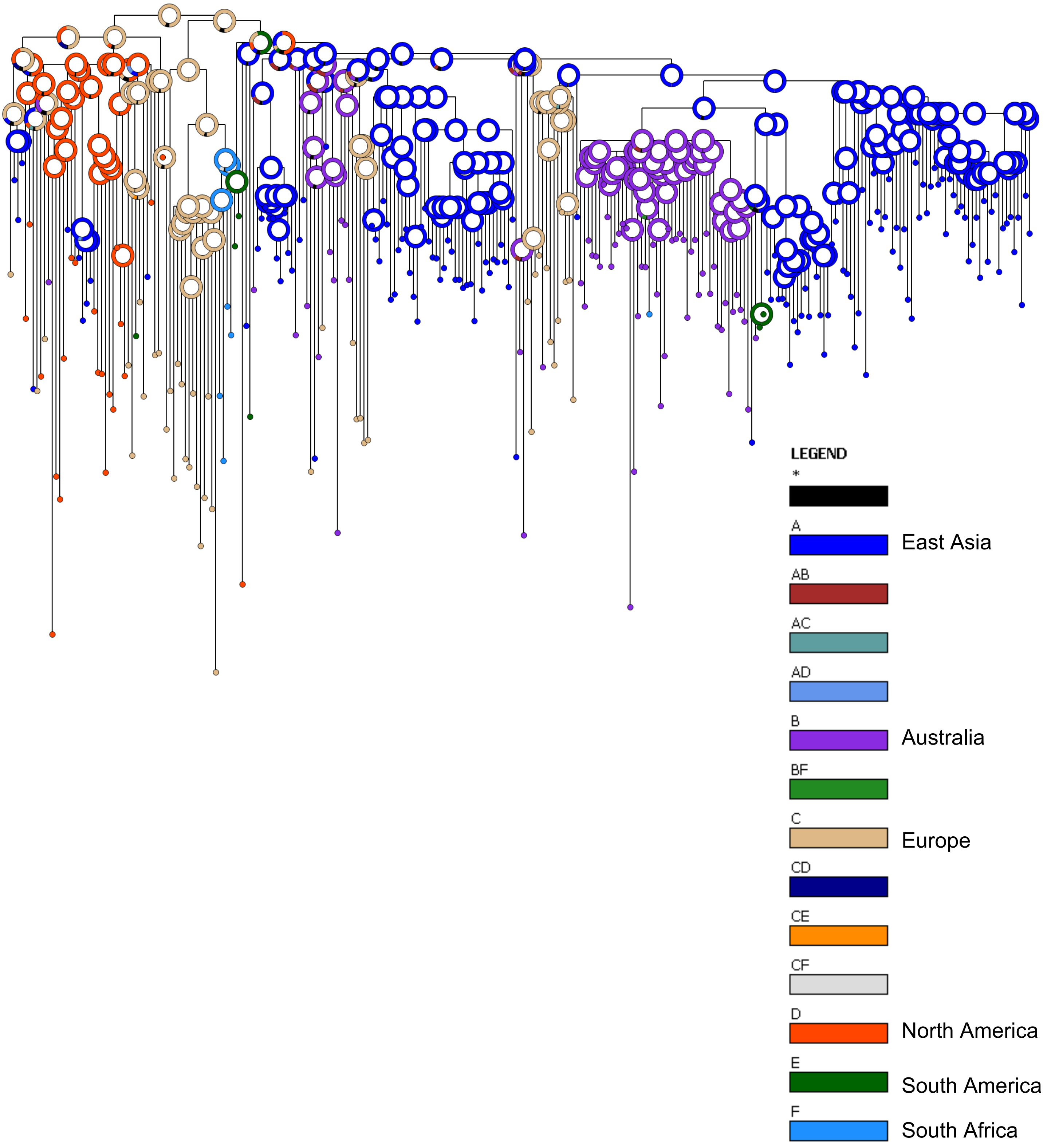

Supplementary Fig. 5. Phylogeographic analysis of the *M. kansasii* main cluster based on the Bayestraits MCMC model.

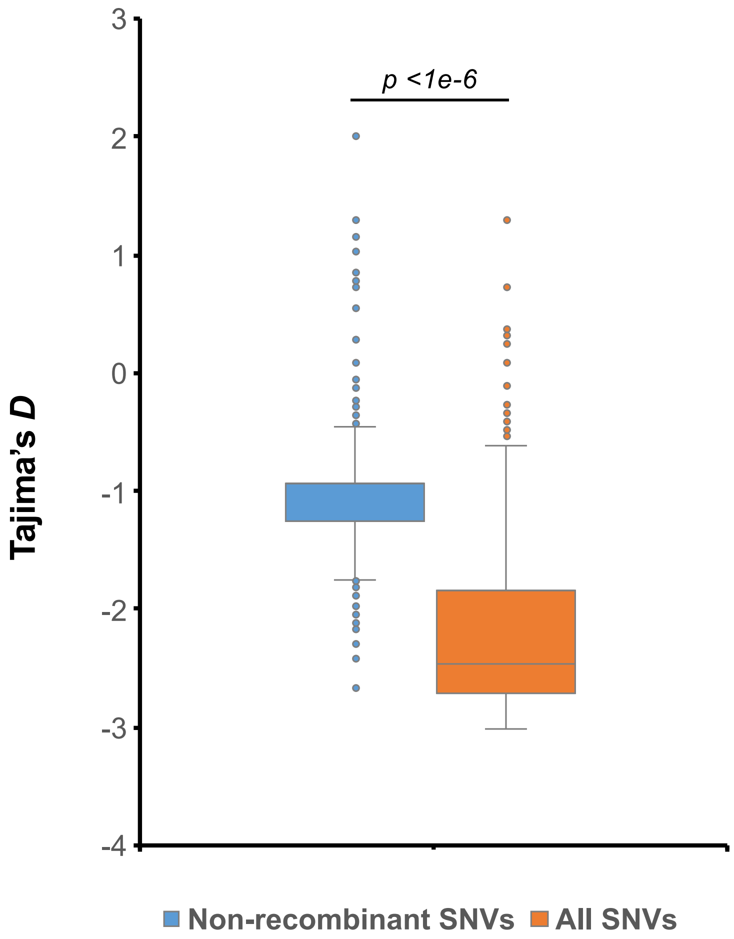

Supplementary Fig. 6. Tajima's *D* statistics of the *M. kansasii* main cluster based on all SNVs or Non-recombinant SNVs. Non-recombinant SNVs, values from 2020 genes; All SNVs, values from 4256 genes. Boxes show the median and interquartile range (IQR) while whiskers extend to a maximum of 1.5× IQR. Significance level is determined by the Mann Whitney test (two-sided). Source data are provided as a Source Data file.

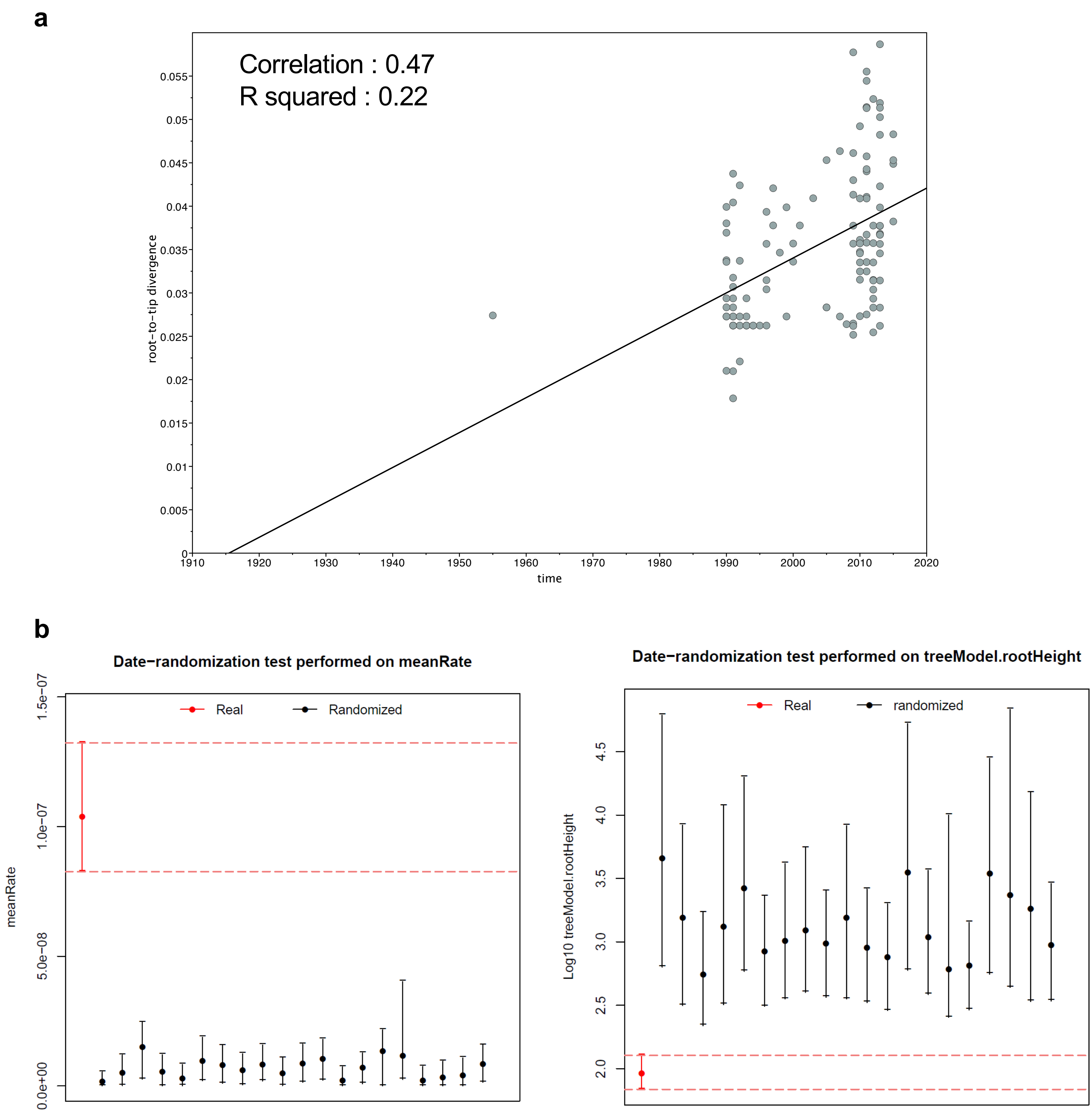

Supplementary Fig. 7. Root-to-tip analysis (**a**) and date-randomization test (**b**) performed on 121 MKMC isolates. For the root-to-tip analysis, a positive correlation (0.47) between genetic divergence and sampling time indicates that the data set is suitable for phylogenetic molecular clock analysis. Source data are provided as a Source Data file. In the date-randomization tests, the 95% confidence intervals (CI) for the estimates of the clock rate (left graph) and root height (right graph) of the observed data (in red) do not overlap with the confidence intervals for the estimates obtained from the randomized sets (in black, 20 independent runs), indicating strong temporal signals. Data are presented as mean and 95% CI.

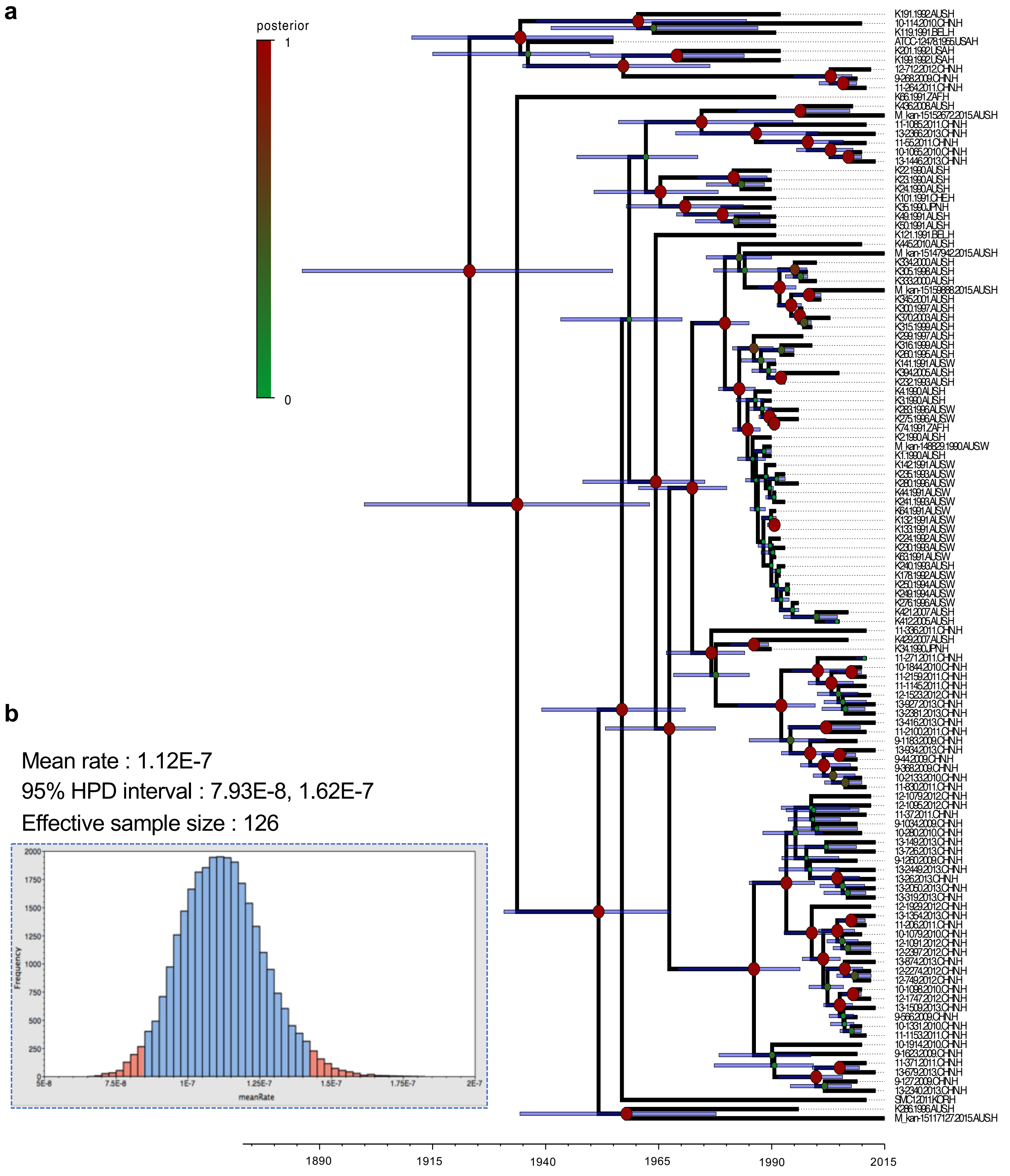

Supplementary Fig. 8. Bayesian evolutionary analysis of 121 MKMC isolates. **a** Maximum clade credibility tree of the 121 isolates. Node bars indicate 95% CI of height. **b** Posterior distribution for the evolutionary rate.

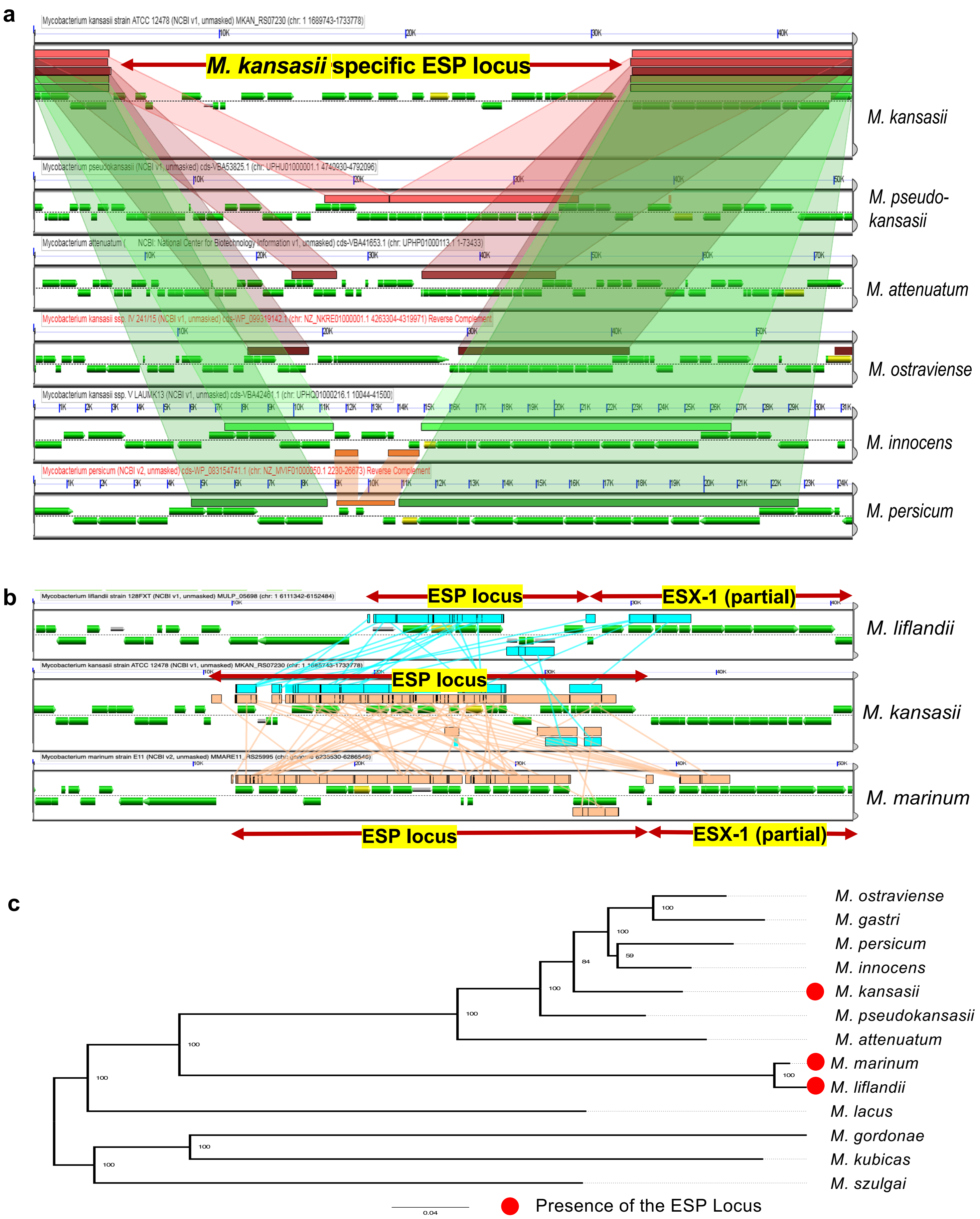

Supplementary Fig. 9. a Synteny map for the ESP locus in the MKC species. **b** Synteny map for the *M. kansasii* specific ESP locus in *M. marinum* and *M. liflandii*. **c** Maximum likelihood phylogeny of the MKC and close related mycobacterium species. Filled red circles indicate presence of the ESP locus.

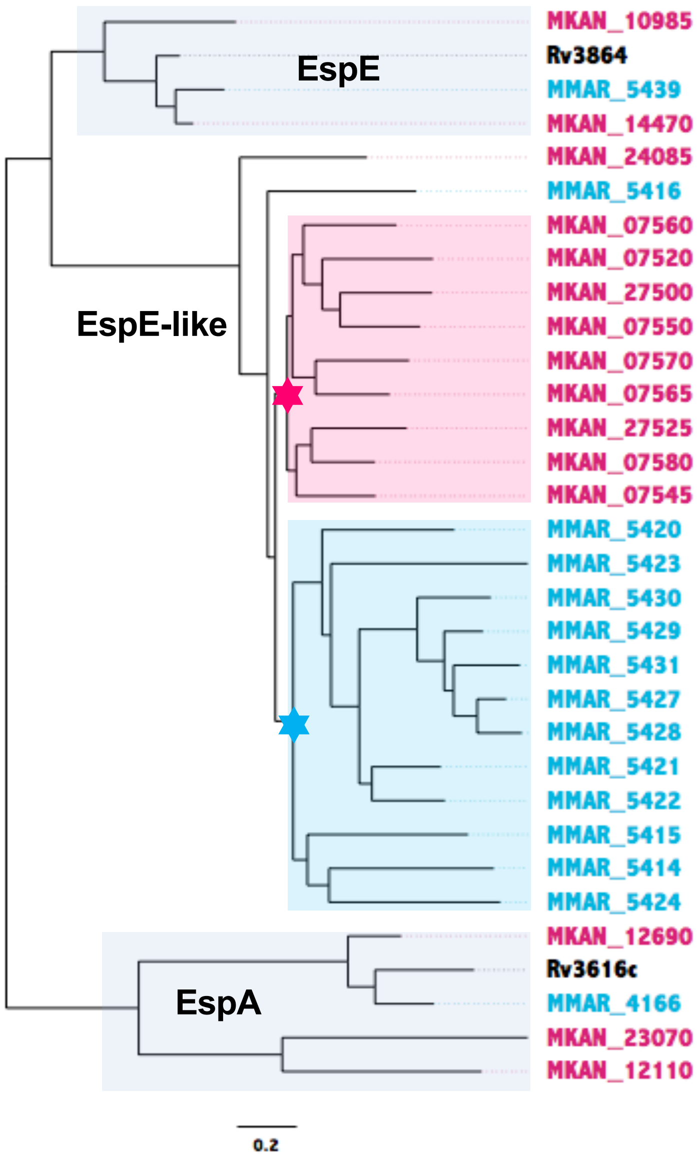

Supplementary Fig. 10. Genetic relationship (Neighbor-Joining phylogeny) between EspE, EspE-like and EspA orthologs of *M. knasaii* (in red), *M. marinum* (in blue) and *M. tuberculosis* (in black). The stars represent potential gene expansions of *espE*-like genes in *M. kansasii* and *M. marinum*.

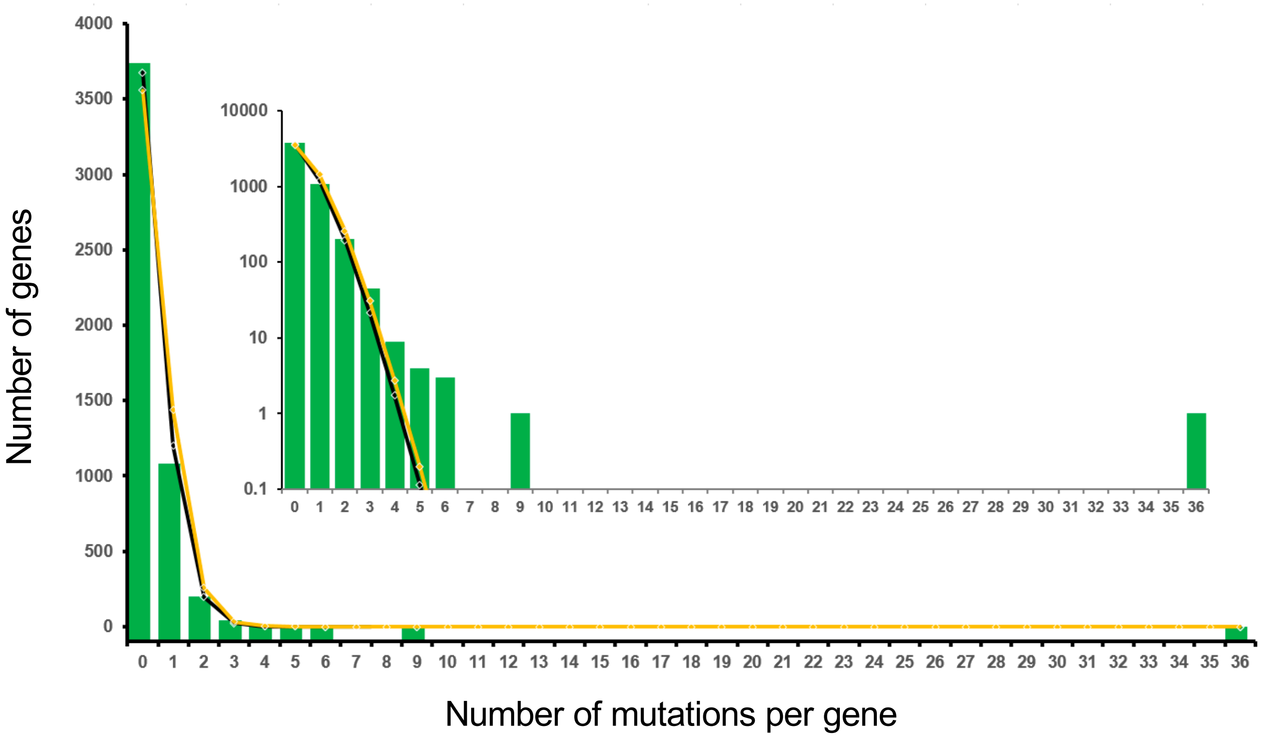

Supplementary Fig. 11. Distribution of number of non-recombinant mutations (SNVs and short indels) per genes. Lines indicate 95% confidence interval of mean predicted values under a Poisson distribution fitted to the data shown in green. Inset shows gene count on a log scale to better show deviations from the Poisson model at high numbers of mutations per gene. Source data are provided as a Source Data file.

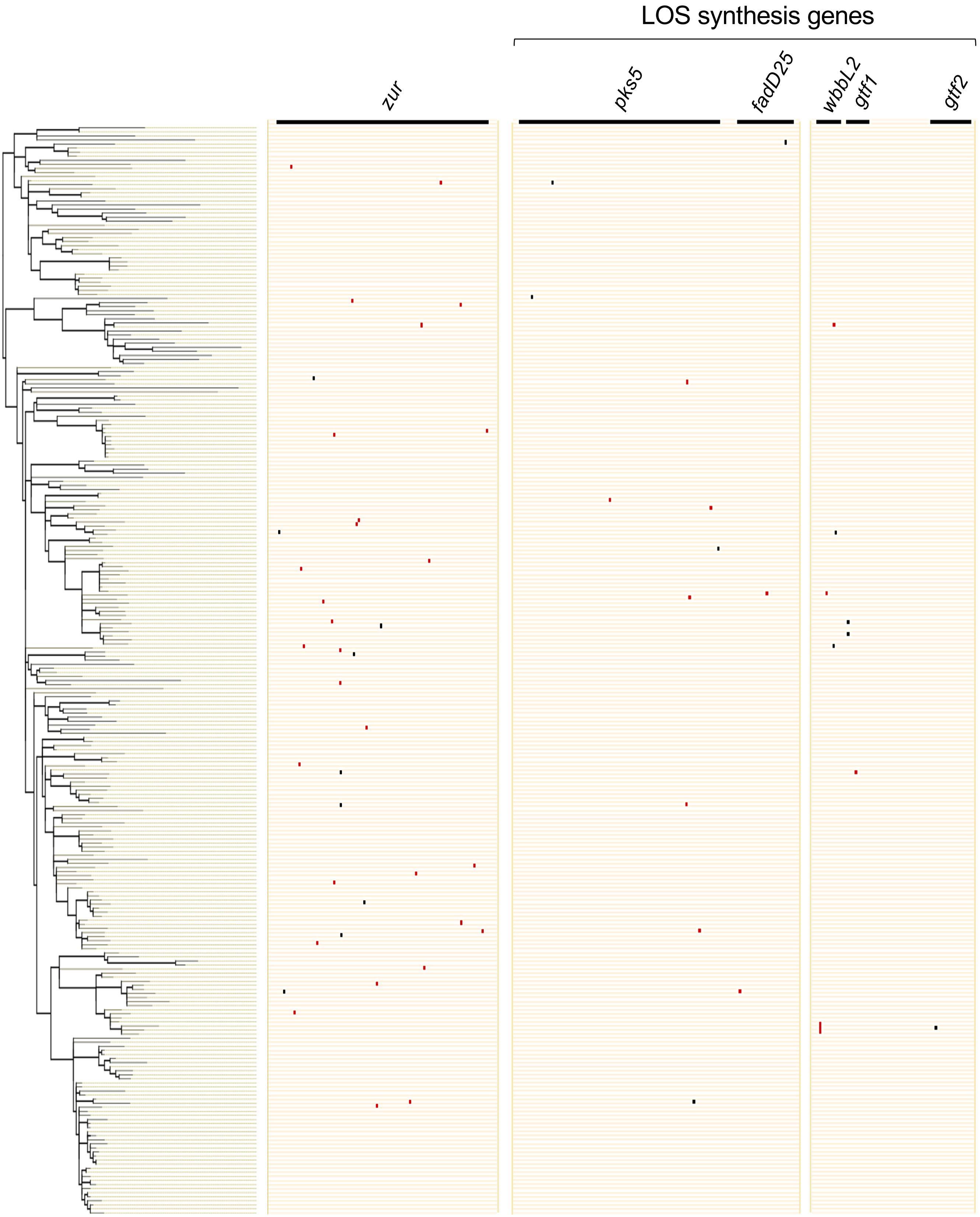

Supplementary Fig. 12. Distribution of non-recombinant homozygous mutations in *zur* and LOS synthesis genes in the MKMC strains and their emergences along the phylogeny. Red, single nucleotide variant; black, insertion or deletion.

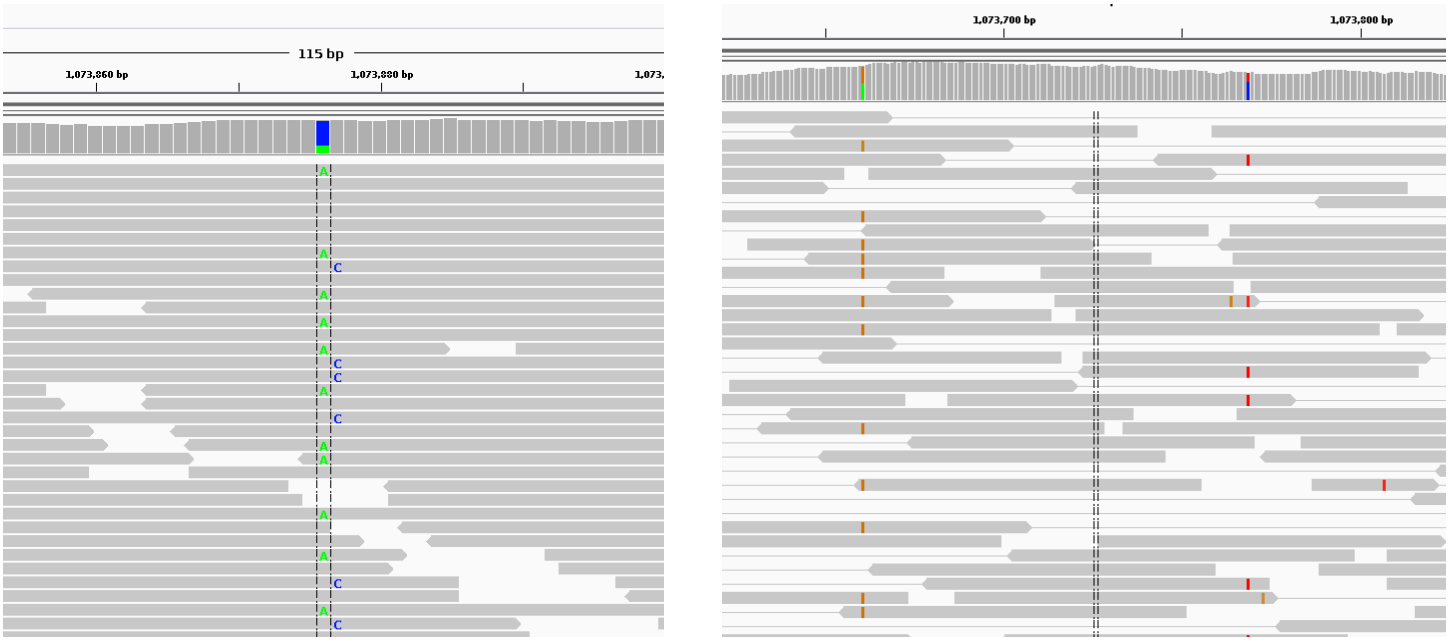

13-874 12-1384

Supplementary Fig. 13. Mapping profiles in *zur* coding region in two representative isolates with double mutations. Grey bands, sequencing reads (paired ends). Mutations are highlighted in colors other than grey.

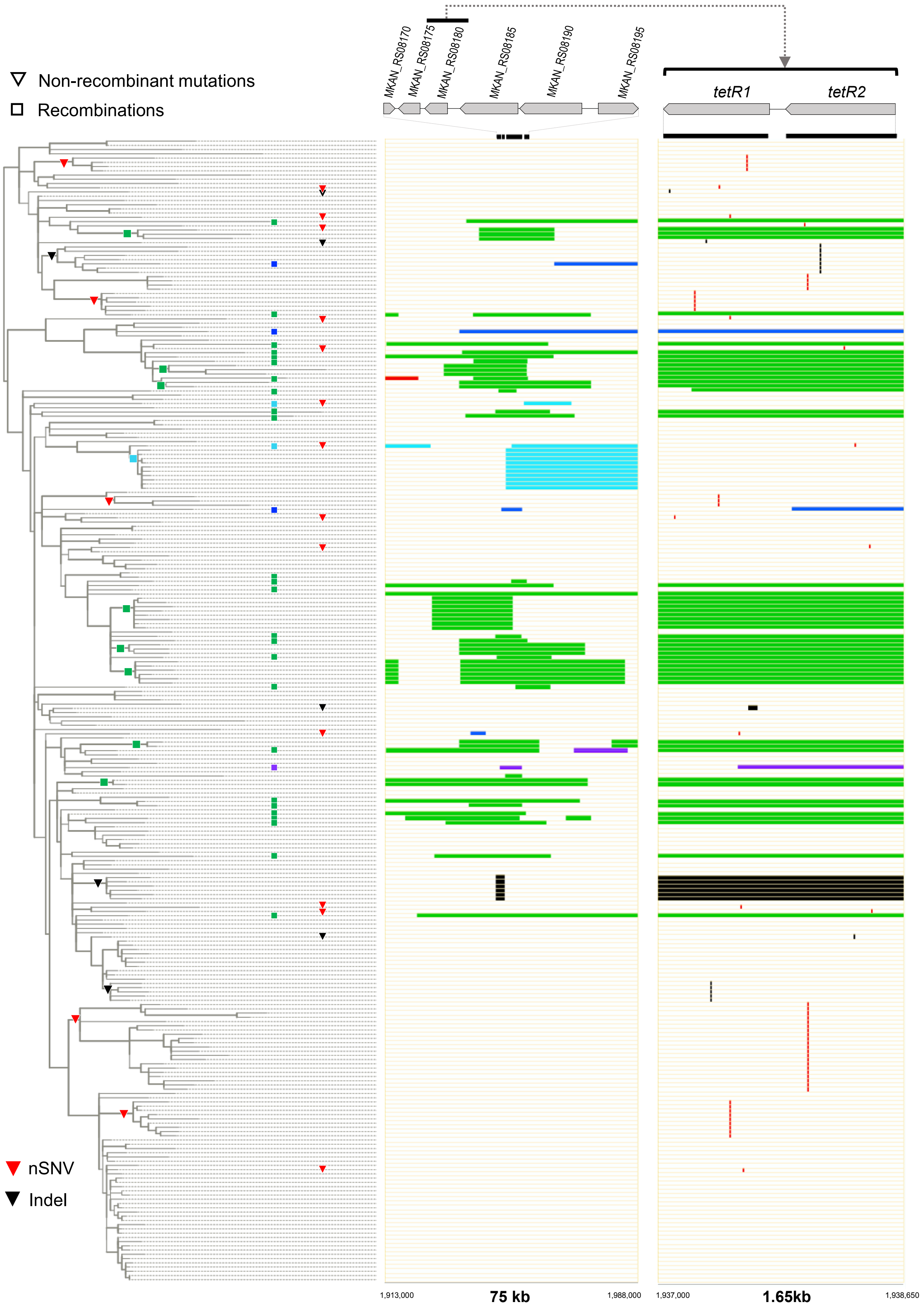

Supplementary Fig. 14. Recombinant fragments and non-recombinant mutations in *tetR1/2* and flanking regions, and mapping of the recombination/mutation events to the maximum-likelihood phylogeny based on the maximum parsimony algorithm. Recombinant fragments are colored according to their donor species as indicated in Figure 2a. Red, nonsynonymous single nucleotide variant (nSNV); black, insertion or deletion (Indel).

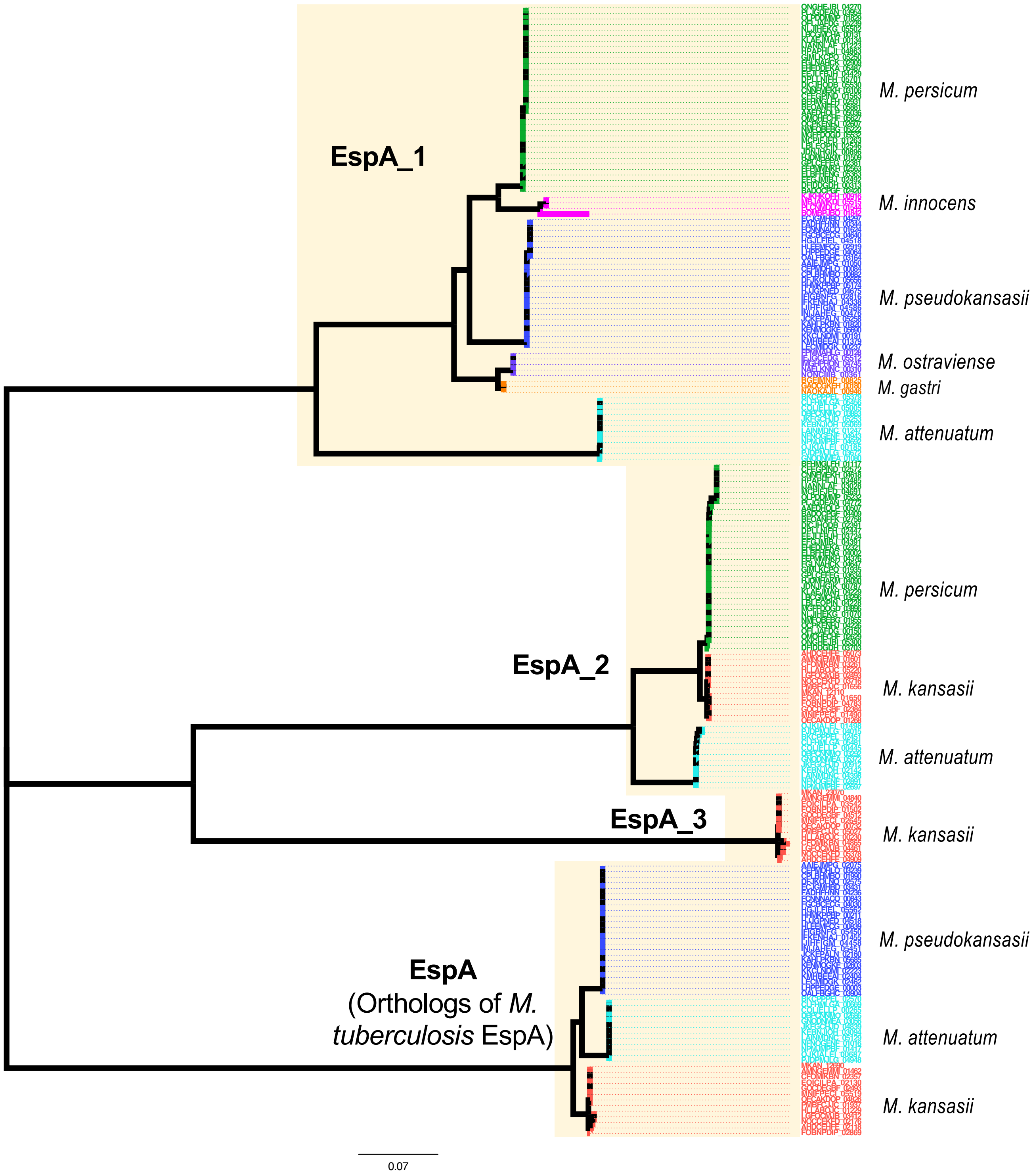

Supplementary Fig. 15. The distribution and genetic relationship (Neighbor-Joining phylogeny) of EspA and its paralogs in *M. kansasii* complex. Tip labels are colored to represent genes from different species.

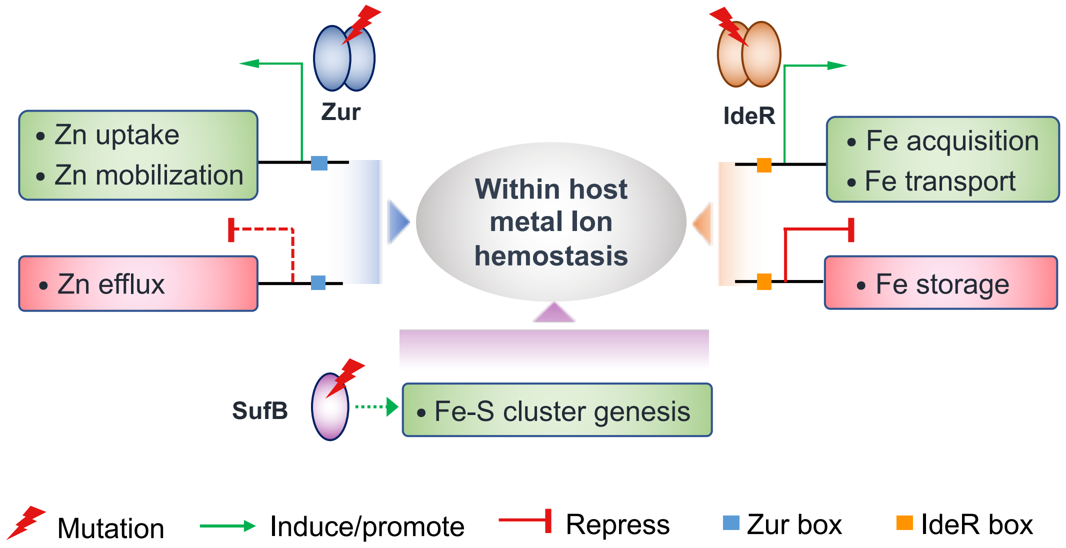

Supplementary Fig. 16. Schematic diagram depicting the potential influence of *zur*, *ideR* and *sufB* mutations on the regulation of zinc and iron homeostasis.

Supplementary Table 1. Prevalence of *M. kansasii* among nontuberculosis mycobacterium infections in different areas of China [a].

Study duration	Province/ city	Method for species identification	Number of NTM isolates	Number of *M. kansasii* isolates	Prevalence of *M. kansasii*	Reference
2004-2009	Shandong	Sequencing of 16S rRNA	68	5	7.35%	1
2008-2011	Heibei	Sequencing of 16s RNA and ITS	95	7	7.37%	2
2011-2013	Hubei	Sequencing of 16s rRNA and ITS	160	2	1.25%	3
2008	Jiangsu	GenoType CM/AS	60	4	6.67%	4
2010-2015	Beijing	Sequencing of 16s rRNA and ITS	232	23	9.91%	5
2005-2012	Fujian	multilocus sequence analysis (*rpoB*, *hsp65* and 16-23s rRNA ITS)	405	1	0.25%	6
2005-2012	Gansu	multilocus sequence analysis (*rpoB*, *hsp65* and 16-23s rRNA ITS)	41	0	0.00%	6
2012-2014	Guangdong	multilocus sequence analysis (*rpoB*, *hsp65*, 16s rRNA and ITS)	938	84	8.96%	7
2011-2014	Zhejiang	Sequencing of *hsp65*	372	36	9.68%	8
2012	Hunan	Biochip	225	3	1.33%	9
2008-2012	Shanghai	Sequencing of 16S rRNA	616	277	44.97%	10

[a] prevalence of *M. kansasii* in global regions outside China mainland was described in supplementary reference #11.

Supplementary Table 2. Mutation profile and morphology of the 12 rough *M. kansasii* clinical isolates and five representative smooth isolates from Shanghai.

Isolate (species)	Mutations in LOS synthesis genes[#]	Morpholgy in L-J slant	Colony morphology in 7H10 plate
9-1623 (*M. kansasii*)	*pks5* Leu369Pro	Rough	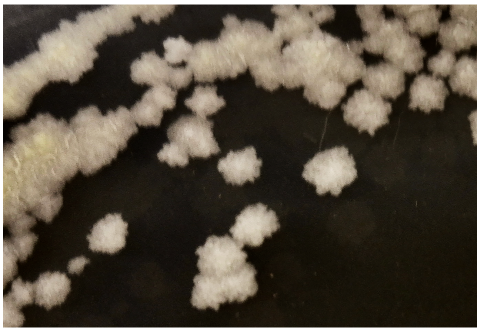
10-280 (*M. kansasii*)	*pks5* Ile735fs	Rough	NA
10-1065 (*M. kansasii*)	*wbbL2* Glu187fs	Rough	NA
10-1079 (*M. kansasii*)	*pks5* Gly230Glu	Rough	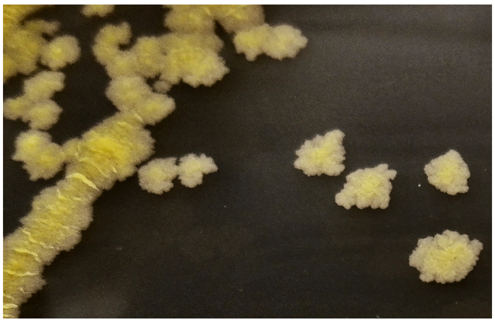
11-55 (*M. kansasii*)	*fadD25* Trp33*<br>*gtf2* Thr184fs	Rough	NA
11-1085 (*M. kansasii*)	*fadD25* Glu288*<br>*wbbL2* Gly91Asp	Rough	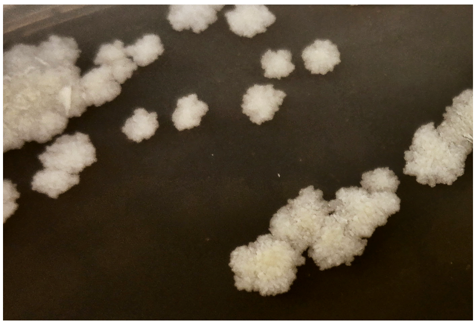

11-1145 (*M. kansasii*)	*fadD25* Ser7Arg & Leu8Ser	Rough	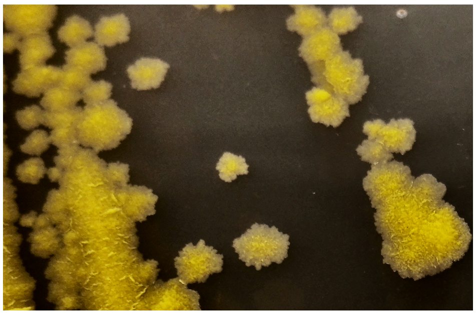
11-2254 (*M. kansasii*)	*fadD25* Val543fs	Rough	NA
12-1384 (*M. kansasii*)	*gtf2* Tyr202His & Cys17Arg	Rought	NA
13-494 (*M. kansasii*)	*gtf1* Ser10fs	Rough	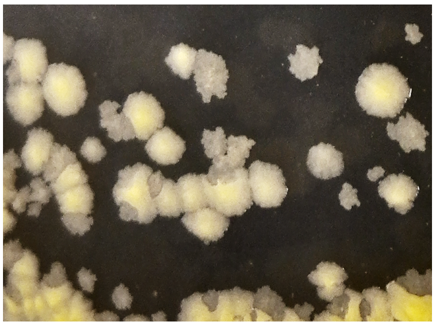
13-934 (*M. kansasii*)	*gtf1* Glu381fs	Rough	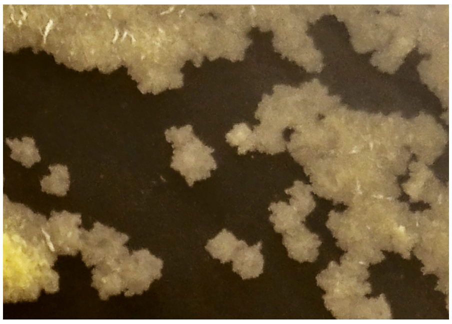
13-2434 (*M. kansasii*)	*pks5* Tyr335His	Rough	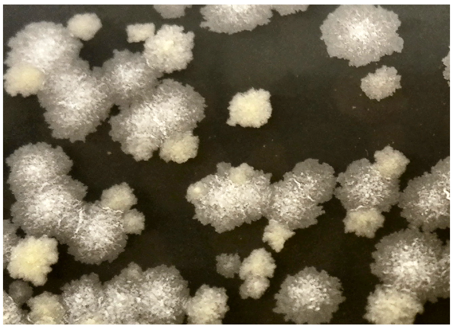

9-1581 (*M. kansasii*)	-	Smooth	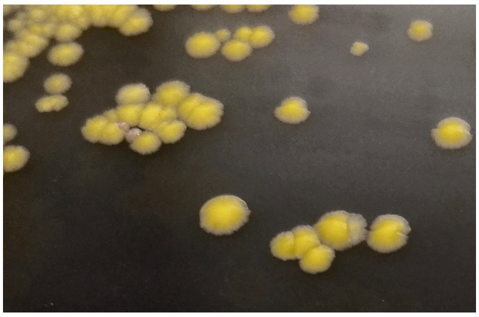
10-114 (*M. kansasii*)	-	Smooth	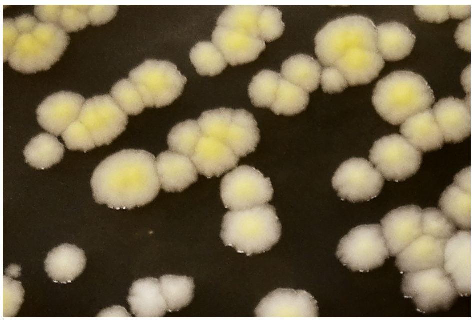
10-1523 (*M. kansasii*)	-	Smooth	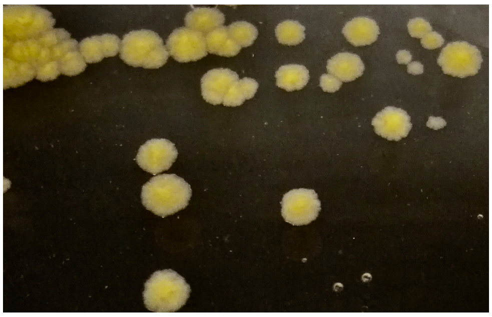
13-2184 (*M. kansasii*)	-	Smooth	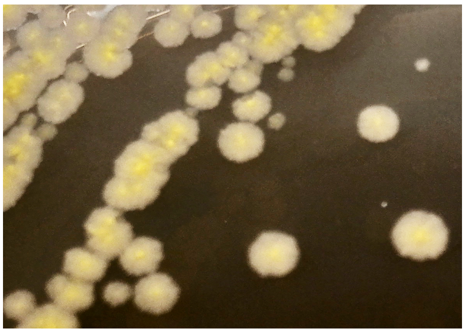
13-2204 (*M. persicum*)	-	Smooth	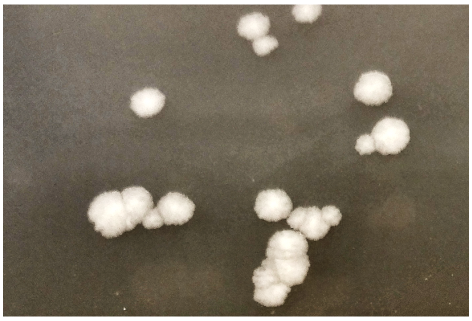

#Underlines indicate unfixed (heterozygous) mutations.

Supplementary References

1. Jing, H. *et al.* Prevalence of nontuberculous mycobacteria infection, China, 2004-2009. *Emerg Infect Dis* **18**, 527-8 (2012).
2. Wang, X. *et al.* Prevalence and drug resistance of nontuberculous mycobacteria, northern China, 2008-2011. *Emerg Infect Dis* **20**, 1252-3 (2014).
3. Yu, X.L. *et al.* Identification and characterization of non-tuberculous mycobacteria isolated from tuberculosis suspects in Southern-central China. *PLoS One* **9**, e114353 (2014).
4. Shao, Y. *et al.* The epidemiology and geographic distribution of nontuberculous mycobacteria clinical isolates from sputum samples in the eastern region of China. *PLoS Negl Trop Dis* **9**, e0003623 (2015).
5. Duan, H. *et al.* Clinical Significance of Nontuberculous Mycobacteria Isolated From Respiratory Specimens in a Chinese Tuberculosis Tertiary Care Center. *Sci Rep* **6**, 36299 (2016).
6. Liu, H. *et al.* Identification of Species of Nontuberculous Mycobacteria Clinical Isolates from 8 Provinces of China. *Biomed Res Int* **2016**, 2153910 (2016).
7. Pang, Y. *et al.* Diversity of nontuberculous mycobacteria in eastern and southern China: a cross-sectional study. *Eur Respir J* **49**, 1601429 (2017).
8. Pan, A. et al. Analysis of pathogen spectrum of nontuberculosis Mycobacteria in Zhejiang province, *Chinese Journal of Health Laboratory Technology* (In Chinese) **25**, 4298-430 (2015)
9. Qi, Z. et al. Gene chips typing and drug sensitivity analysis of 225 cases of non-tuberculous mycobacteria. *Journal of clinical pulmonary medicine* (In Chinese) **19**, 105-107 (2014).
10. Wu, J. *et al.* Increase in nontuberculous mycobacteria isolated in Shanghai, China: results from a population-based study. *PLoS One* **9**, e109736 (2014).
11. Hoefsloot, W. *et al.* The geographic diversity of nontuberculous mycobacteria isolated from pulmonary samples: an NTM-NET collaborative study. *Eur Respir J* **42**, 1604-13 (2013).
