# Supplementary material for: Population genomics provides insights into the evolution and adaptation to humans of the waterborne pathogen Mycobacterium kansasii
Source: Nat Commun. 2021 May 3;12:2491. doi: 10.1038/s41467-021-22760-6 (PMC8093194; doi:10.1038/s41467-021-22760-6)
Supplement: Supplementary file 3 — Description of Additional Supplementary Files [file 41467_2021_22760_MOESM3_ESM.pdf]

Description of Additional Supplementary Files

File Name: Supplementary Data 1
Description: Metadata for the isolates included in this study.

File Name: Supplementary Data 2
Description: Information for the 146 genes specific to M. kansasii.

File Name: Supplementary Data 3
Description: Non-synonymous mutations in genes under potential positive selection.
