# Supplementary material for: Population genomics provides insights into the evolution and adaptation to humans of the waterborne pathogen Mycobacterium kansasii
Source: Nat Commun. 2021 May 3;12:2491. doi: 10.1038/s41467-021-22760-6 (PMC8093194; doi:10.1038/s41467-021-22760-6)
Supplement: Supplementary file 7 — Reporting Summary [file 41467_2021_22760_MOESM7_ESM.pdf]

Corresponding author(s): Qian Gao, Tim P. Stinear, Marcel A. Behr, Qichao Pan, Xinchun Chen, Yanlin Zhao, Tao Luo

Last updated by author(s): Mar 1, 2021

# Reporting Summary

Nature Research wishes to improve the reproducibility of the work that we publish. This form provides structure for consistency and transparency in reporting. For further information on Nature Research policies, see our Editorial Policies and the Editorial Policy Checklist.

## Statistics

For all statistical analyses, confirm that the following items are present in the figure legend, table legend, main text, or Methods section.

| n/a | Confirmed | |
|---|---|---|
| ☐ | ☒ | The exact sample size ($n$) for each experimental group/condition, given as a discrete number and unit of measurement |
| ☒ | ☐ | A statement on whether measurements were taken from distinct samples or whether the same sample was measured repeatedly |
| ☐ | ☒ | The statistical test(s) used AND whether they are one- or two-sided<br>*Only common tests should be described solely by name; describe more complex techniques in the Methods section.* |
| ☒ | ☐ | A description of all covariates tested |
| ☒ | ☐ | A description of any assumptions or corrections, such as tests of normality and adjustment for multiple comparisons |
| ☐ | ☒ | A full description of the statistical parameters including central tendency (e.g. means) or other basic estimates (e.g. regression coefficient) AND variation (e.g. standard deviation) or associated estimates of uncertainty (e.g. confidence intervals) |
| ☐ | ☒ | For null hypothesis testing, the test statistic (e.g. $F$, $t$, $r$) with confidence intervals, effect sizes, degrees of freedom and $P$ value noted<br>*Give P values as exact values whenever suitable.* |
| ☐ | ☒ | For Bayesian analysis, information on the choice of priors and Markov chain Monte Carlo settings |
| ☒ | ☐ | For hierarchical and complex designs, identification of the appropriate level for tests and full reporting of outcomes |
| ☒ | ☐ | Estimates of effect sizes (e.g. Cohen's $d$, Pearson's $r$), indicating how they were calculated |

*Our web collection on statistics for biologists contains articles on many of the points above.*

## Software and code

Policy information about availability of computer code

| | |
|---|---|
| Data collection | SRA Toolkit (v2.10.8) |
| Data analysis | Trimmomatic (v0.30), SPAdes (v3.13.1), Quast (v5.02), fastANI (v1.1), Roary (v3.11.2), Prokka (v1.14.6), MAFFT (v7.407), RaxML (v8.2.12), SplitsTree (v4.14.5), PopGenome (v2.7.5), hierBAPS, fastGEAR, Gubbins (v2.3.4), Minimap2 (v2.17), Phandango (v1.1.0), Bowtie2 (2.3.5), SAMtools (v1.9), VarScan (v1.4.3), Network (v5.0), RASP (v4.0), TempEst (v1.5.3), TipDatingBeast (v1.1-0), BEAST (v1.8.0), Tracer (v1.6), TimeTree (v0.6.4), iTOL (v5.7), ClicO FS (v1.0), BLAST (v2.9.0) |

For manuscripts utilizing custom algorithms or software that are central to the research but not yet described in published literature, software must be made available to editors and reviewers. We strongly encourage code deposition in a community repository (e.g. GitHub). See the Nature Research guidelines for submitting code & software for further information.

## Data

Policy information about availability of data

All manuscripts must include a data availability statement. This statement should provide the following information, where applicable:

- Accession codes, unique identifiers, or web links for publicly available datasets
- A list of figures that have associated raw data
- A description of any restrictions on data availability

Sequence data associated with this study was deposited in the Sequence Read Archive (SRA) of NCBI under project accession PRJNA323639 (https://www.ncbi.nlm.nih.gov//bioproject/PRJNA323639). Accessions for publicly available genomic data are given in the Supplementary Data 1.

# Field-specific reporting

Please select the one below that is the best fit for your research. If you are not sure, read the appropriate sections before making your selection.

- [ ] Life sciences
- [ ] Behavioural & social sciences
- [x] Ecological, evolutionary & environmental sciences

For a reference copy of the document with all sections, see nature.com/documents/nr-reporting-summary-flat.pdf

# Ecological, evolutionary & environmental sciences study design

All studies must disclose on these points even when the disclosure is negative.

| | |
|---|---|
| Study description | Population genomics analysis of 358 M. kansasii complex (MKC) isolates obtained from global water and clinical sources. Insights has been gained in evolution, infection and host adaptation of M. kansasii |
| Research sample | Pulmonary disease by M. kansasii has been reported globally. Municipal water is believed to be the major reservoir for clinical M. kansasii infection. We collected 358 isolates of M. kansasii complex from global clinical and city water sources to study the global diversity , evolution and host-adaptation of this pathogen. |
| Sampling strategy | Sample size was determined through comparison with published population genomic studies about the global diversity of other mycobacterium, including M. tuberculosis (Comas, I. et al. Nat Genet 2013, N=259), M. leprea (Benjak, A. et. al. Nat Commun 2018, N=154). The aim of the sampling efforts was to collect samples from as many global areas as possible to represent global diversity of M. kansasii complex. We finally collected 358 isolates from 18 countries in current study. |
| Data collection | Isolates were collected from water or clinical sources by the authors. Isolate metadata was recorded by the authors. Colony morphology was recorded by authors of Shanghai CDC and Fudan University. Genome sequencing was done by commercial company (HaploX Biotech. Co., Shenzhen, China) using Illumina Hiseq 2000 or NextSeq 500. |
| Timing and spatial scale | Isolates were collected from 1953-2018 at the following frequencies: 1953 1; 1968 1; 1990 14; 1991 31; 1992 9; 1993 5; 1994 2; 1995 1; 1996 5; 1997 6; 1998 2; 1999 4; 2000 2; 2001 2; 2003 4; 2004 2; 2005 3; 2007-2008 40; 2009 18; 2010 28; 2011 32; 2012 31; 2013 36; 2014 2; 2015 25; 2016 1; 2018 1; NA 45. Isolates are collected from 18 countries in 6 areas: East Asia 160; Oceania 74; Europe 80; North America 27; South America 12; Africa 5. We consider our collection as a broad overview of M. kansasii from global areas over 76 years, which is suitable for study global diversity and Bayesian Evolutionary Analysis based on tip-date calibration. |
| Data exclusions | No data was excluded |
| Reproducibility | We performed two independent runs for recombination prediction by fastGEAR or Gubbins. Three runs of BEAST were performed to assure independent convergence of the chains. |
| Randomization | Bacterial isolates were randomly collected. Bioinformatic analysis of all samples was done simultaneously to ensure consistency of procession. |
| Blinding | Blinding was not relevant here as no direct comparisons or conclusions being drawn between how samples were treated. |

Did the study involve field work? [ ] Yes [x] No

# Reporting for specific materials, systems and methods

We require information from authors about some types of materials, experimental systems and methods used in many studies. Here, indicate whether each material, system or method listed is relevant to your study. If you are not sure if a list item applies to your research, read the appropriate section before selecting a response.

## Materials & experimental systems

| n/a | Involved in the study | |
|---|---|---|
| [x] | [ ] | Antibodies |
| [x] | [ ] | Eukaryotic cell lines |
| [x] | [ ] | Palaeontology and archaeology |
| [x] | [ ] | Animals and other organisms |
| [x] | [ ] | Human research participants |
| [x] | [ ] | Clinical data |
| [x] | [ ] | Dual use research of concern |

## Methods

| n/a | Involved in the study | |
|---|---|---|
| [x] | [ ] | ChIP-seq |
| [x] | [ ] | Flow cytometry |
| [x] | [ ] | MRI-based neuroimaging |
